# Small ceramide tames big p53 mutant beast

**DOI:** 10.18632/oncotarget.27738

**Published:** 2020-09-15

**Authors:** Ronald A. Hill, Zhijun Liu, Yong-Yu Liu

**Keywords:** ceramide, p53 tumor suppressor, missense mutation, nanomicelle, drug resistance

**Figure 1:**
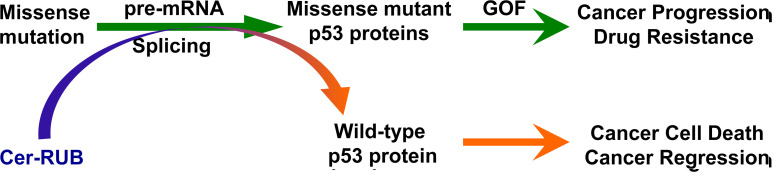
p53 mutant proteins contribute to oncogenic gain-of-function (GOF) that promotes cancer progression and emergence of drug resistance. Cer-RUB nanomicelles restore wild-type p53 in cancer cells, thus inducing cancer cell death and contributing to resultant cancer regression.

The p53 tumor suppressor plays key roles not only in preventing tumorigenesis, but also in combating cancer progression. Missense p53 mutants are commonly detected in cancers and their gain-of-functions (GOF) promote cancer progression. Wild-type p53 (wt-p53) protein is encoded from the *TP53* gene, and serves as a guardian of genome integrity. p53 triggers transcription and expression of p53-responsive genes, including p21, Bax and Puma, thereby promoting cancer cell death and accordant cancer regression (Figure 1). GOF resulting from p53 mutant proteins that are encoded from *TP53* missense mutations promotes cancer progression and causes emergent resistance to cancer chemotherapies [1] (Figure 1). Codons 175, 248, and 273 are referred to as mutation “hotspots”, and missense mutations at these loci are prevalently and commonly detected in cancers of ovaries, colon and lungs [1]. Restoration of wt-p53 protein expression and function in conjunction with ablation of mutants thus holds great promise for devising efficacious anticancer therapies [2, 3]. Ceramides (Cer), which are sphingolipids, can act as cellular signals to induce cancer cells to apoptosis, proliferation-arrest or autophagy [4]. Recently, small Cer-RUB nanomicelles were identified as an agent for restoring p53 tumor suppression and overcoming drug resistance as well as cancer progression in ovarian cancer cells and tumors that carry p53 missense G248Q mutation [5].

Cer-RUB nanomicelles effectively delivered a C_6_-ceramide (C_6_-Cer; a synthetic Cer comprised of six carbons in the fatty acyl chain attached to sphingosine) into cells and into tissues when administered to mice. C_6_-Cer is more potent than physiological long-carbon chain ones (C_12–24_-Cers) for killing cancer cells. Exogenously delivered C_6_-Cer not only increases levels of endogenous Cers, but also displays greater potency in inducing cell death. C_6_-Cer is more slowly glucosylated (by glucosylceramide synthase, GCS), and may be converted more slowly to the corresponding sphingosine-1-phosphate (S1P) by deacylation (ceramidases) followed by phosphorylation (sphingosine kinases) [5–7]. By exploiting these two prominent diversion pathways, metastatic cancers can suppress free Cer levels, and furthermore, mount resistance to anticancer drugs via elevated glucosylceramide and S1P [4, 7]. We adopted the natural and digestible glycoside rubusoside (RUB), rather than any of the synthetic polymers that are commonly exploited for the purpose (e.g., polyethylene glycol [PEG], polyethylenimine [PEI], poly[lactic-coglycolic acid] [PLGA], graphene oxide), to encapsulate C_6_-Cer and generate small uniform Cer-RUB nanomicelles (~32 nm). The nanomicelles exhibited greatly increased water-solubility *vs.* C_6_-Cer, unquestionably contributing to improved bioavailability to cells in culture and to tissues of mice. We did not detect any significant nonspecific toxicity of RUB in cell culture or animal studies, even at significantly higher concentrations or amounts than with the RUB-encapsulated Cer at the highest doses used. Pharmacokinetic studies further indicate Cer-RUB nanomicelles can effectively deliver C_6_-Cer into tumors and other tissues for various prospective therapeutic purposes, when administered intraperitoneally or orally [5, 8].

The Cer-RUB nanomicelles are much more effective than C_6_-Cer delivered alone in sensitizing cancer cells or tumors carrying p53 missense mutations to therapeutic agents, and display high potential to be developed as a selective treatment for p53-mutant cancers. Previous studies indicated that either exogenous C_6_-Cer provided or endogenous Cer generated by suppression of Cer glycosylation restored wt-p53 protein and p53-dependent tumor suppression in ovarian cancer cells carrying deletion-mutations in region coding exon-5 of the p53 DNA-binding domain (18-bp deleted in NCI/ADR-RES cells, 21-bp deleted in OVCAR-8 cells) [3, 9]. OVCAR-3 cancer cells *homozygously* carry *TP53* R248Q missense mutation and are resistant to many anticancer drugs, including cisplatin [5]. Cer-RUB comparably delivered C_6_-Cer into OVCAR-3 and A2780 ovarian cancer cells (wt-p53), or xenograft tumors generated therefrom. Significantly, namomicellular Cer-RUB proved much more efficacious, upon concomitant treatment with cisplatin, in killing OVCAR-3 cells and ovarian cancer stem cells (CD24^+^/CD133^+^), and in inhibiting growth of corresponding xenograft tumors, than was the case with A2780 cells or tumors [5]. Upon further assessment, the re-sensitizing effects of Cer-RUB nanomicelles correlated with restored protein levels of wt-p53 (phosphorylated at Ser15, pp53) and of p53-responsive genes (p21, Bax). Further, Cer-RUB nanomicelles also restored the levels of pp53 and p21 in transgenic mice carrying the p53 R172H/^+^ missense mutation [5]. The evidence accrued so far demonstrates that Cer-RUB nanomicelles can be applied to restore p53-based tumor suppression in cancers carrying the deletion-mutation, and hotspot missense mutation.

Although intriguing, more and more evidence supports a truth that Cer-RUB nanomicelles or Cers are involved in restoring p53-dependent tumor suppression activity in cancer cells carrying p53 mutations, most likely via modulating pre-mRNA splicing in post-transcription processes. Based on experimental evidence, mutant cancer cells can generate pre-mRNA transcripts molecules, including wt and mutant [3, 10]. Prior studies showed that C_6_-Cer activate protein phosphatase-1, whence dephosphorylated serine/arginine-rich splicing-factor 1 (SRSF1) is translocated to the nucleus, in turn promoting pre-mRNA splicing to preferentially produce wt-p53 mRNA and protein [3, 9]. Most likely, Cer modulates posttranscriptional processes to restore wt-p53 expression in OVCAR-3 cells carrying *TP53* R248Q mutation, though the mechanisms still need to be further detailed. *N*^6^-methyladenosine (m^6^A) generated at mutant codon, or m^6^As formed close to mutant codons, also can alter the affinity of splicing factors and then spliceosome binding favoring mutant p53 pre-mRNA to produce missense mutant [10]. Regardless, the Cer-RUB nanomicelles, which enable delivery of Cer, even by oral route, to efficaciously restore p53-dependent tumor suppression in a case where p53 mutations are extant, and reverse emergent chemotherapy resistance, while sparing normal tissues. Cer-RUB nanomicells hold great promise for improving treatment outcomes of cancers, notably for refractory and recurrent cases where prognoses are otherwise poor.
